# Supplementary material for: Association between oral microbiome and five types of respiratory infections: a two-sample Mendelian randomization study in east Asian population
Source: Front Microbiol. 2024 Apr 10;15:1392473. doi: 10.3389/fmicb.2024.1392473 (PMC11039966; doi:10.3389/fmicb.2024.1392473)
Supplement: Supplementary file 1 [file Data_Sheet_1.zip › Supplementary Materials/MR plots of saliva/Chronic sinusitis/s__Tannerella_sp002890585_mgs_2607/forest.pdf]

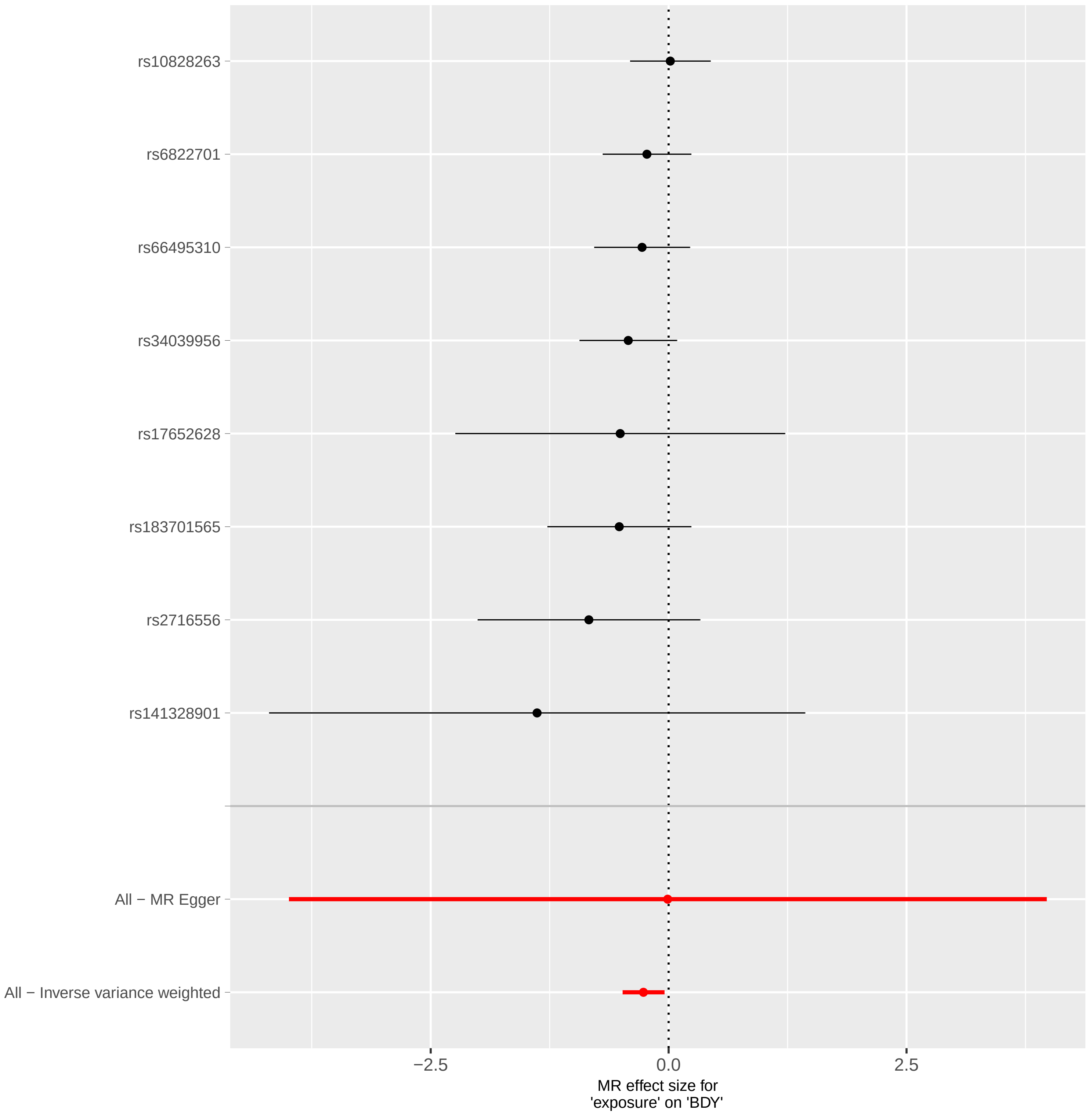
rs10828263
rs6822701
rs66495310
rs34039956
rs17652628
rs183701565
rs2716556
rs141328901
All − MR Egger
All − Inverse variance weighted
−2.5
0.0
2.5
MR effect size for
'exposure' on 'BDY'
